# Systemic anti‐cancer therapy associated with the occurrence of peripheral neurotoxicity and, specifically, peripheral neuropathy

**DOI:** 10.1002/ijc.70414

**Published:** 2026-03-05

**Authors:** Cassie Higgins, Lynn R. Gauthier, Blair H. Smith, Lesley Colvin

**Affiliations:** ^1^ Division of Population Health and Genomics School of Medicine, University of Dundee, Mailbox 1, Level 7, Corridor L, Ninewells Hospital Dundee UK; ^2^ Department of Family and Emergency Medicine, Faculty of Medicine Université Laval; Centre de Recherche du CHU de Québec‐Université Laval, Centre intégré de cancérologie, CHU de Québec‐Université Laval Québec City Quebec Canada

**Keywords:** cancer, chemotherapy, peripheral neuropathy, peripheral neurotoxicity, systemic anti‐cancer therapy

## Abstract

The investigation of peripheral neurotoxicity associated with systemic anticancer therapy (SACT) agents is often confined to a small range of chemotherapy agents. This study aimed to identify all SACT agents associated with peripheral neurotoxicity and, specifically, peripheral neuropathy, and to provide incidence estimates for the development of each type of neurotoxicity associated with each agent. Antineoplastic agents approved globally for clinical and/or research purposes were identified through triangulation of nine national and global drug product databases. The class of each agent was identified using DrugBank Online. Evidence of peripheral neurotoxicity and, specifically, peripheral neuropathy was obtained by reviewing Micromedex (and, where required, supplemented by data reported in national clinical trials registries). A total of 467 approved antineoplastic agents were identified for clinical and/or research purposes globally. Peripheral neurotoxicity (including neuropathy) was associated with 144 (31%) agents: 49 (45%) classical chemotherapeutic agents; 61 (21%) targeted therapies; 26 (63%) immunotherapies; and 8 (25%) hormone therapies. Peripheral neuropathy, specifically, was associated with 77 (16%) agents: 30 (27%) classical chemotherapeutic agents; 31 (11%) targeted therapies; 16 (39%) immunotherapies; and no hormone therapies. Based on the currently available evidence, this inventory of neurotoxic SACT agents could be considered exhaustive. From clinical and research perspectives, a comprehensive inventory of neurotoxic SACT agents is pivotal to informing drug design and understanding and implementing appropriate drug choice, as well as investigating the mechanisms involved in the neurotoxic effects of agents and identifying neuroprotective strategies for patients undergoing SACT.

## Graphical abstract

What's new?

While the effectiveness of systemic anticancer therapy is well documented, it commonly causes severe toxicity. This study of the 467 systemic anticancer therapy agents currently approved globally for clinical and/or research purposes found that peripheral neurotoxicity is associated with 45% of classical chemotherapies, 21% of targeted therapies, 63% of immunotherapies, and 25% of hormone therapies. Almost two‐thirds of the classical chemotherapies and immunotherapies associated with peripheral neurotoxicity were specifically associated with peripheral neuropathy, versus a fifth of the targeted therapies. The findings may inform the clinical planning of systemic anticancer therapy interventions and the further investigation of their neurotoxic effects.

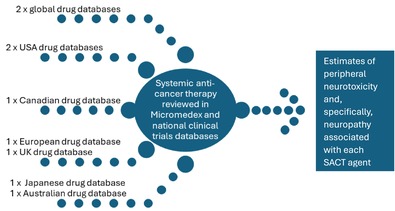

**Abbreviations**
ABPI: Association of the British Pharmaceutical Industry
CTLA‐4: cytotoxic t‐lymphocyte associated protein 4
LAG‐3: Lymphocyte‐activation gene 3
NCI: National Cancer Institute
NIH: National Institutes of Health
NSW: New South Wales
PD‐1: programmed cell death protein 1
PR China: People's Republic of China
SACT: systemic anticancer therapy
UK: United Kingdom
USA: United States of America

## 1 INTRODUCTION

Systemic anti‐cancer therapy (SACT) is a mainstay of cancer treatment. Whilst its effectiveness in treating cancer is well documented, it commonly causes severe toxicity. Neurotoxicity can be severe enough during treatment to require dose reductions or withdrawal, compromising the chance of remission and survival. It can also substantially reduce quality of life during treatment and in the longer term. For some, neurotoxicity may resolve quickly; however, it is estimated to persist in at least 30% of people at six‐month follow‐up.^1^ Studies investigating the neurotoxic complications of SACT have tended to focus their attention on several specific classes of agent (i.e., some of the platinum derivatives, taxanes, vinca alkaloids, proteasome inhibitors and thalidomide analogues) to the exclusion of others.^2^ To ensure patient safety and to inform future medical research, we need to identify all potentially neurotoxic SACT agents, including those in more recently developed treatment modalities, such as targeted therapies, hormone therapies and immunotherapies.

Classical chemotherapy is cytotoxic, acting at different cell cycle phases with the aim of inhibiting cancer cell division, but these agents may also harm healthy central or peripheral neurones directly or indirectly by causing, for example, inflammation or damage to glial cells.^3^ However, there is mounting evidence to suggest that mechanisms beyond those underlying antiproliferative effects could result in structural harm, mitochondrial dysfunction and proinflammatory cytokine release,^4^, ^5^, ^6^, ^7^, ^8^ necessitating a review of the potential neurotoxic complications associated with all SACT agents. Indeed, there is evidence of neurotoxic effects associated with other classical chemotherapies,^9^ targeted therapies,^10^ hormone therapies^11^, ^12^ and immunotherapies.^13^, ^14^ Whilst the central nervous system is afforded some degree of protection from the physical (tight junctions) and metabolic (enzyme) aspects of the blood–brain barrier,^15^ the peripheral nervous system has no such protection and is particularly vulnerable to the effects of potentially toxic drugs. Therefore, it is important to understand which SACT agents are associated with peripheral neurotoxicity and, specifically, neuropathy, which is considered the most prevalent and dose‐limiting peripheral neurotoxicity.^16^

We aimed to identify all potentially neurotoxic SACT agents by, first, compiling a list of all SACT agents approved globally for clinical and/or research purposes and, secondly, recording evidence of associated peripheral neurotoxicity and, specifically, neuropathy.

## 2 METHODS

### 2.1 Identification and classification of SACT agents

The Association of the British Pharmaceutical Industry (ABPI) provides global rankings of the number of clinical trials initiated in 2021, by country, by phase.^17^ Relevant data were sought from all 12 countries identified by the ABPI, and data were provided for 11 (see below). No response was received from the National Medical Products Administration (PR China). Through triangulation of data from the following nine drug databases, two of which provide global data, SACT agents approved for clinical and/or research purposes were identified:

1. USA: National Institutes of Health (NIH) National Cancer Institute (NCI)^18^
2. USA: American Cancer Society^19^
3. Canada: Government of Canada: Health Canada^20^
4. Spain, Germany, France, Poland, Italy and Belgium (and other European nations): European Medicines Agency^21^
5. UK: Cancer Research UK^22^
6. Japan: National Cancer Centre Hospital^23^
7. Australia: eviQ, NSW Government^24^
8. Global: World Health Organization^25^
9. Global: George Pantziarka TP53 Trust^26^

Each of the agents was classified by its treatment modality (classical chemotherapy, targeted therapy, immunotherapy or hormone therapy). To ensure consistency, classifications were identified using DrugBank Online.^27^

### 2.2 Identification of peripheral neurotoxicity and neuropathy associated with SACT agents

Evidence of peripheral neurotoxicity and, specifically, neuropathy was obtained through reviewing each agent in Micromedex,^28^ which compiles data reported in clinical trials. Micromedex is a tertiary resource, curated from primary literature using an accredited editorial process and is updated daily. The method of using Micromedex for this purpose has been used previously^29^ to investigate which of the commonly used classical chemotherapies are associated with neurotoxicity. Very occasionally, where adverse effects were not reported for specific agents in Micromedex, national clinical trials registers were reviewed and relevant data were extracted.

Peripheral neurotoxicity, defined according to diseases, signs and symptoms triangulated from three sources,^30^, ^31^, ^32^ is described in Table S1 and included, for example, dysaesthesia, paraesthesia, and muscle weakness.

### 2.3 Presentation of findings

A complete record of study findings is contained in the Supporting Information: classical chemotherapies (Table S2); targeted therapies (Table S3); immunotherapies (Table S4); and hormone therapies (Table S5). Agents associated with peripheral neuropathy and/or neurotoxicity are reported in the Section 3.

Whilst many SACT agents are commonly combined in protocols, for clarity, the findings of the present study describe peripheral neurotoxicity/neuropathy associated with each single agent, with a few exceptions. Firstly, two dyads are used uniquely together: trifluridine + tipiracil and tegafur + uracil are reported in the antimetabolite section. Secondly, cedazuridine is used in combination with decitabine; decitabine alone is reported in the antimetabolite section, and the dyad is reported in the targeted therapies section. Thirdly, the tegafur + gimeracil + oteracil triad is reported in the targeted therapies section. Fourthly, the three LAG‐3 checkpoint inhibitors are each used in combination with one of the PD‐1 checkpoint inhibitors: nivolumab (PD‐1) + relatlimab (LAG‐3); pembrolizumab (PD‐1) + favezelimab (LAG‐3); and cemiplimab (PD‐1) + fianlimab (LAG‐3). The PD‐1 checkpoint inhibitors are reported separately in the appropriate section, and the three dyads are reported in the LAG‐3 checkpoint inhibitor section. Fifthly, prolgolimab is reported in the PD‐1 inhibitors section, both on its own and in combination with Nurulimab (CTLA‐4 inhibitor).

Some agents could be listed within more than one drug class (e.g., mitoxantrone acts as both a topoisomerase II inhibitor and an anthracycline cytotoxic antibiotic) or within more than one therapeutic modality (e.g., some monoclonal antibodies, which are targeted therapies, also act as immunotherapies). Where this is the case, agents are listed only once.

Where data are available in Micromedex, the estimated occurrence of peripheral neuropathy following treatment is reported for each agent. Where the estimated incidence is reported as not known (‘nk’) in this article, this indicates that the agent is associated with peripheral neuropathy but that the incidence is not yet known.

## 3 RESULTS

A total of 467 SACT agents were identified: 110 classical chemotherapeutic agents; 284 targeted therapies; 41 immunotherapies; and 32 hormone therapies. Oncolytic viruses, used as immunotherapies, were not included in this review because there is no systematic way in which to collate information on their neurotoxic effects.

Peripheral neurotoxicity (including neuropathy) was associated with 144 (31%) agents: 49 (45%) classical chemotherapeutic agents; 61 (21%) targeted therapies; 26 (63%) immunotherapies; and 8 (25%) hormone therapies.

Peripheral neuropathy, specifically, was associated with 77 (16%) agents: 30 (27%) classical chemotherapeutic agents; 31 (11%) targeted therapies; 16 (39%) immunotherapies; and no hormone therapies. These agents are listed in Table 1, by treatment modality, along with incidence estimates, where available, of the development of peripheral neuropathy following treatment.

**TABLE 1:** SACT agents associated with the development of peripheral neuropathy.

1a: Classical chemotherapeutic agents (estimated incidence of peripheral neuropathy following treatment)		
*Alkylating agents: Cisplatin and derivatives* Cisplatin (61%) Oxaliplatin (69–92%) *Alkylating agents: Nitrosoureas* Fotemustine (n/k) Semustine (n/k) Treosulfan (n/k) *Alkylating agents: Other* Altretamine (≤31%) Lurbinectedin (11%) Procarbazine (n/k) *Alkylating agents: Tetrazines* Dacarbazine (n/k)	*Cytotoxic antibiotics: Anthracyclines* Aclarubicin (n/k) Amrubicin (1%) *Cytotoxic antibiotics: Non‐anthracyclines* Utidelone (23%) *Antimetabolites* Gemcitabine (15–64%) Nelarabine (12–21%) *Mitotic inhibitors (anti‐microtubule agents): Taxanes* Cabazitaxel (7–18%) Docetaxel (4–30%) Nab‐paclitaxel (71%) Paclitaxel (60%) Paclitaxel poliglumex (19%)	*Mitotic inhibitors (anti‐microtubule agents): Vinca alkaloids* Vincristine (33%) Vindesine (n/k) Vinorelbine (20%) Vintafolide (20%) *Mitotic inhibitors (anti‐microtubule agents): Others* Eribulin (29–35%) Ixabepilone (62–65%) *Proteasome inhibitors* Bortezomib (30–54%) Carfilzomib (<20%) Ixazomib (32%) *Topoisomerase I inhibitors* Belotecan (6%) Etirinotecan pegol (3–7%)

1b: Targeted therapy agents (estimated incidence of peripheral neuropathy following treatment)		
*Monoclonal antibodies (mAbs): Naked* Alemtuzumab (n/k) Cetuximab (45%) Daratumumab (47%) Dinutuximab (9%) Elotuzumab (27%) Isatuximab (54%) Margetuximab (16%) Naxitamab (25–32%) Pertuzumab (6–42%) Retifanlimab (n/k) Toripalimab (30%) Zolbetuximab (≥15%) *Monoclonal antibodies (mAbs): Bispecific* Margetuximab (16%)	*Monoclonal antibodies (mAbs): Antibody‐drug conjugates* Brentuximab vedotin (45–81%) Enfortumab vedotin (50–67%) Fam‐trastuzumab deruxtecan‐nxki (13%) Mirvetuximab soravtansine (33–37%) Polatuzumab vedotin (40–53%) Tisotumab vedotin (11–38%) Trastuzumab duocarmazine (26%)	*Small molecule: Protein kinase inhibitors* Encorafenib (12–62%) Lorlatinib (34–47%) Mirdametinib (21%) Palbociclib (13%) Ponatinib (6–33%) Repotrectinib (49%) *Small molecule: Other* Adagrasib (20%) Decitabine + cedazuridine (4–13%) Plitidepsin (n/k) Suramin (≤40%) Tegafur + gimeracil + oteracil (≥10%)

1c: Immunotherapy agents (estimated incidence of peripheral neuropathy following treatment)		
*Checkpoint inhibitors: PD‐1 inhibitors* Cemiplimab (11–23%) Dostarlimab (64%) Nivolumab (≤12%) Pembrolizumab (1–67%) Tislelizumab (n/k) *Checkpoint inhibitors: LAG‐3 inhibitors* Nivolumab + Relatlimab (<1%)	*Checkpoint inhibitors: PD‐L1 inhibitors* Atezolizumab (33–56%) Avelumab (n/k) Durvalumab (61%) *Checkpoint inhibitors: CTLA‐4 inhibitors* Ipilimumab (2%) Tremelimumab (n/k)	*Cytokines: Interferons* Peginterferon alfa‐2a (<1%) Peginterferon Alfa‐2b (23%) *Other immune system modulators* Bacillus Calmette‐Guérin (n/k) Pomalidomide (17–18%) Thalidomide (10–54%)

*Note*: Incidence estimates as reported in Micromedex; ‘n/k’ indicates that the agent was reported to be associated with peripheral neuropathy but that the incidence estimate was not know.

Peripheral neurotoxicity (other than peripheral neuropathy) was associated with: 19 (17%) classical chemotherapeutic agents; 30 (11%) targeted therapies; 10 (25%) immunotherapies; and 8 (25%) hormone therapies. These agents are listed in Table 2, by treatment modality, along with incidence estimates, where available, of the development of each type of peripheral neurotoxicity following treatment.

**TABLE 2:** SACT agents associated with the development of peripheral neurotoxicity other than peripheral neuropathy.

2a: Classical chemotherapeutic agents: Nature of peripheral neurotoxicity (estimated incidence of peripheral neurotoxicity following treatment)	
*Alkylating agents: Nitrogen mustards* Ifosfamide: Neurotoxicity (15%) *Alkylating agents: Nitrosoureas* Carmustine: Asthenia (22%) *Alkylating agents: Other* Pipobroman: Muscle cramps (n/k) *Cytotoxic antibiotics: Anthracyclines* Valrubicin: Asthenia (4%) *Topoisomerase I inhibitors (camptothecins)* Irinotecan: Asthenia (58–76%) Topotecan: Asthenia (3–25%) *Topoisomerase II inhibitors* Amsacrine: Paraesthesia (n/k)	*Antimetabolites* Azacitidine: Asthenia (n/k) Capecitabine: Paraesthesia (12–21%) Cladribine: Neurotoxicity (n/k) Decitabine: Asthenia (15%) Fludarabine: Asthenia (9%–65%) and neurotoxicity (n/k) Fluorouracil: Neurotoxicity (n/k) Methotrexate: Neurotoxicity (n/k) Pentostatin: Asthenia (10–12%) and neurotoxicity (1–11%) Raltitrexed: Asthenia (2–5%) Trifluridine + tipiracil: Asthenia (52%) *Mitotic inhibitors (anti‐microtubule agents): Vinca alkaloids* Vinblastine: Neurotoxicity (n/k) Vinflunine: Asthenia (n/k)

2b: Targeted therapy agents: Nature of peripheral neurotoxicity (estimated incidence of peripheral neurotoxicity following treatment)	
*Small molecule: Protein kinase inhibitors* Axitinib: Asthenia (21%) Entrectinib: Dysesthesia (34%) Everolimus: Asthenia (13–33%) Fedratinib: Asthenia (n/k) Lazertinib: Paraesthesia (35%) Nilotinib: Asthenia (14%–16%) Regorafenib: Asthenia (n/k) Sunitinib: Asthenia (22%–34%) Temsirolimus: Asthenia (51%) *Small molecule: Other* Arsenic trioxide: Paraesthesia (33%) Asthenia (23%) and paraesthesia (6%) Bexarotene: Asthenia (20–45%) Ivosidenib: Guillain‐Barre syndrome (<1%) Mifamurtide: Asthenia (13%) Olaparib: Asthenia (n/k) Pixantrone: Asthenia (≥10%) Romidepsin: Asthenia (n/k) Rucaparib: Asthenia (n/k) Selinexor: Neurotoxicity (25%–30%)	*Monoclonal antibodies (mAbs): Naked* Mogamulizumab: Guillain–Barré syndrome (n/k) Rituximab: Asthenia (26%) *Monoclonal antibodies (mAbs): Bispecific* Blinatumomab: Neurotoxicity (65%) Elranatamab: Neurotoxicity (59%) Glofitamab: Neurotoxicity (n/k) Mosunetuzumab: Neurotoxicity (39%) Talquetamab: Neurotoxicity (55%) Tarlatamab: Neurotoxicity (47%) Teclistamab: Neurotoxicity (15%) *Other protein‐based therapies* Asparaginase: Neurotoxicity (n/k) Crisantaspase: Neurotoxicity (n/k)

2c: Immunotherapy agents: Nature of peripheral neurotoxicity (estimated incidence of peripheral neurotoxicity following treatment)	
*Chimeric antigen receptor (CAR) T‐cell therapy* Axicabtagene ciloleucel (‘axi‐cel’): Neurotoxicity (78%) Ciltacabtagene autoleucel (‘cilta‐cel’): Neurotoxicity (24–26%) Idecabtagene vicleucel (‘ide‐cel’): Neurotoxicity (28%) Obecabtagene autoleucel (‘obe‐cel’): Neurotoxicity (64%) Tisagenlecleucel (‘tisa‐cel’): Neurotoxicity (43–71%)	*Cytokines: Interleukins* Aldesleukin (‘Interleukin‐2’ or ‘IL‐2’): Asthenia (23%) *Cytokines: Interferons* Interferon alfa (IFN‐alpha): Paraesthesia (13%) and asthenia (n/k) *Checkpoint inhibitors: PD‐1 + CTLA‐4 inhibitors* Prolgolimab + nurulimab: Asthenia (10%) *Other immune system modulators* Lenalidomide: Asthenia (≤30%) Tretinoin: Paraesthesia (17–26%)

2d: Hormone therapy agents: Nature of peripheral neurotoxicity (estimated incidence of peripheral neurotoxicity following treatment)	
*Selective oestrogen receptor degraders (SERDs)* Fulvestrant: Asthenia (6%) *Aromatase inhibitors (AIs)* Anastrozole: Asthenia (16–19%) Letrozole: Asthenia (4–34%) *Luteinizing hormone‐releasing hormone (LHRH) agonists (also called LHRH analogues)* Leuprolide: Asthenia (8–18%) *Androgenic hormones* Fluoxymesterone: Paraesthesia (n/k)	*Anti‐androgens (i.e., androgen receptor antagonists): First generation* Bicalutamide: Asthenia (22%) *Anti‐androgens (i.e., androgen receptor antagonists): Second generation* Enzalutamide: Asthenia (24–51%) and cauda equina syndrome (n/k) *Adrenolytics* Mitotane: Neurotoxicity (n/k)

*Note*: Incidence estimates as reported in Micromedex; ‘n/k’ indicates that the agent was reported to be associated with peripheral neuropathy but that the incidence estimate was not know.

Summing the data from Tables 1 and 2 shows that the proportion of agents associated with the development of any peripheral neurotoxicity (including peripheral neuropathy) differed by treatment modality: 49 (45%) classical chemotherapeutic agents; 61 (21%) targeted therapies; 26 (63%) immunotherapies; and 8 (25%) hormone therapies.

## 4 DISCUSSION

These findings show that 467 SACT agents are currently approved for clinical and/or research purposes globally. Peripheral neurotoxicity was associated with almost a half of classical chemotherapies, a fifth of targeted therapies, almost two‐thirds of immunotherapies and a quarter of hormone therapies. Furthermore, almost two‐thirds of the classical chemotherapies and immunotherapies that are associated with peripheral neurotoxicity were associated specifically with the occurrence of peripheral neuropathy, compared with only a fifth of the targeted therapies. The preponderance of targeted therapies currently under investigation, particularly protein kinase inhibitors and other small molecule drugs, may account for this latter finding – as this field of research expands, the proportion of targeted therapies associated with peripheral neurotoxicity and, specifically, neuropathy may increase.

Where SACT agents were associated with peripheral neurotoxicity other than neuropathy, common adverse effects included asthenia and unspecified neurotoxicity. Firstly, this latter descriptor is potentially problematic—it suggests that many studies have reported associations with peripheral neurotoxicity but not specified the nature of these neurotoxic effects, which may potentially have included peripheral neuropathy. Secondly, whilst there is no current consensus on the definition of peripheral neuropathy, the descriptors used to characterise neurotoxicity in the absence of peripheral neuropathy (e.g., paraesthesia, dysaesthesia and asthenia) could indicate the potential presence of peripheral neuropathy in some cases. It is, therefore, possible that the number of agents identified as being associated specifically with peripheral neuropathy may be an underestimate. A second limitation relates to the potential under‐reporting of harms in clinical trials. Commonly, the principal aim of clinical trials is focused on progression‐free survival as the primary outcome, with adverse effects frequently identified as a secondary outcome, which could lead to inaccurate recording of these events. Additionally, Seruga and colleagues^33^ propose several other reasons for the under‐reporting of harms in relation to anticancer drugs, such as disparities in clinician and patient evaluations of adverse events, the under‐reporting of harms influenced by clinicians or sponsors and short‐term follow‐up failing to detect longer‐term and potentially serious toxicities. Despite these limitations in previously published studies, the present study provides a more comprehensive account of peripheral neurotoxicity and neuropathy associated with SACT agents than has been published to date.

The findings have potential value to clinicians when planning SACT interventions and to researchers aiming to investigate the neurotoxic effects of such treatment. Systematic reviews and meta‐analyses provide high quality evidence, informing clinical guidelines, and the design of their search strategies must ensure the inclusion of all available evidence. Given that many clinical trials specify the agent under investigation, rather than using generic SACT‐related terms, it is important that search terms for systematic reviews can include the names of all neurotoxic agents, and this study provides such a list.

## AUTHOR CONTRIBUTIONS

**Cassie Higgins:** Conceptualization; data curation; investigation; formal analysis; methodology; writing – original draft. **Lynn R. Gauthier:** Data curation; investigation; methodology; writing – review and editing. **Blair H. Smith:** Methodology; writing – review and editing. **Lesley Colvin:** Conceptualization; methodology; writing – review and editing.

## CONFLICT OF INTEREST STATEMENT

Cassie Higgins, Lynn R. Gauthier and Blair H. Smith have no relevant conflicts of interest to disclose. Lesley Colvin discloses the following potential conflicts of interest: SenseCheQ @ Home—early community diagnosis of Chemotherapy‐Induced Peripheral Neuropathy. Role: Local Principal Investigator. ESPSRC (£1,991,210). PharRmacoepidemiology Of NEuropathic Pain (PRONE): Identifying predictors of neuropathic pain medication prescribing and response in diabetic peripheral neuropathy and chemotherapy‐induced peripheral neuropathy. British Journal of Anaesthesia/RCoA PhD studentship (£97,636). Mapping the Complexity of Pain: SenseCheQ: Community‐based sensory testing for early identification of Chemotherapy Induced Peripheral Neuropathy. Role: Local Principal Investigator. UKRI/ versus Arthritis/ Eli Lilly: APDP (£1,004,693). Consortium bid: Partnership for Assessment and Investigation of Neuropathic Pain: Studies Tracking Outcomes, Risks and Mechanisms. Role: Principal Investigator for CIPN component. UKRI/ versus Arthritis/ Eli Lilly: APDP (£3,454,155). Scottish Intercollegiate Guideline Network (SIGN), Healthcare Improvement Scotland Evidence Directorate: Vice Chair of SIGN Council and Chair of the Guideline Development Group for SIGN Guideline on Management of Chronic Pain. Royal College of Anaesthetists (Scottish Board): Co‐opted member for Scottish Intercollegiate Guideline Network (SIGN).

## Supporting information


**Table S1:** Diseases, signs and symptoms indicave of the presence of peripheral neurotoxicity.
**Table S2:** Classical chemotherapeuc agents (*N* = 110).
**Table S3:** Targeted therapy approved for specific types of cancer (*N* = 284).
**Table S4:** Immunotherapy by class of drug (*N* = 41).
**Table S5:** Hormone therapy by class of drug (*N* = 32).

## Data Availability

Only publicly available data were used in this study, and data sources and handling of these data are described in the Supporting Information. Further information is available from the corresponding author upon request.
