# Supplementary material for: Systemic anti‐cancer therapy associated with the occurrence of peripheral neurotoxicity and, specifically, peripheral neuropathy
Source: Int J Cancer. 2026 Mar 5;159(2):460–6. doi: 10.1002/ijc.70414 (PMC13193447; doi:10.1002/ijc.70414)
Supplement: Supplementary file 1 — Table S1: Diseases, signs and symptoms indicave of the presence of peripheral neurotoxicity. Table S2: Classical chemotherapeuc agents (N = 110). Table S3: Targeted therapy approved for specific types of cancer (N = 284). Table S4: Immunotherapy by class of drug (N = 41). Table S5: Hormone therapy by class of drug (N = 32). [file IJC-159-460-s001.pdf]

Systemic anti-cancer therapy associated with the occurrence of peripheral neurotoxicity and, specifically, peripheral neuropathy

Cassie Higgins, Lynn R Gauthier, Blair H Smith, Lesley Colvin

Table of contents

Item	Page
Table S1: Diseases, signs and symptoms indicative of the presence of peripheral neurotoxicity	1
Table S2: Classical chemotherapeutic agents (N=110)	1
Table S3: Targeted therapy approved for specific types of cancer (N=284)	4
Table S4: Immunotherapy by class of drug (N=41)	11
Table S5: Hormone therapy by class of drug (N=32)	12

Table S1: Diseases, signs and symptoms indicative of the presence of peripheral neurotoxicity

Definition of peripheral neurotoxicity: diseases, signs and symptoms	
• Ataxia	• Painful sensory or axonal neuropathy
• Autonomic syndrome/dysautonomia	• Paraesthesia
• Brachial plexopathy	• Radiculopathy
• Cranial nerve palsy	• Raynaud syndrome
• Dysaesthesia (mouth, throat, face and hands)	• Sensory, motor and/or autonomic peripheral neuropathy
• Hearing loss	• Taste and smell disorders
• Lhermitte's sign	• Tinnitus
• Motor weakness	• Toxic optic neuropathy
• Muscle weakness, cramps or spasms	• Vocal cord paralysis
• Orthostatic hypotension	

Table S2: Classical chemotherapeutic agents (N=110)

Action	Agent	Evidence of peripheral neurotoxicity	
		**Any peripheral neurotoxicity**	**Peripheral neuropathy specifically**
	Busulfan	No	No

Alkylating agents: Alkyl sulfonates	Mannosulfan	No	No
	Treosulfan	Yes	Yes (N/K)
Alkylating agents: Aziridines	Diaziquone	No	No
	Mitomycin	No	No
Alkylating agents: Cisplatin and derivatives	Carboplatin	No	No
	Cisplatin	Yes	Yes (61%)
	Oxaliplatin	Yes	Yes (69-92%)
	Polyplatillen	No	No
	Satraplatin	No	No
Alkylating agents: Epoxides	Etoglucid	No	No
	Mitobronitol	No	No
Alkylating agents: Ethylene imines	Carboquone	No	No
	Triaziquone	No	No
	Thiotepa	No	No
Alkylating agents: Nitrogen mustard analogues	Bendamustine	No	No
	Chlorambucil	No	No
	Chlormethine	No	No
	Cyclophosphamide	No	No
	Ifosfamide	Neurotoxicity (15%)	No
	Mechlorethamine	No	No
	Melphalan	No	No
	Melphalan flufenamide	No	No
	Prednimustine	No	No
	Trofosfamide	No	No
Alkylating agents: Nitrosoureas	Carmustine	Asthenia (22%)	No
	Fotemustine	Yes	Yes (N/K)
	Lomustine	No	No
	N-Nitroso-N-methylurea	No	No
	Nimustine	No	No
	Ranimustine	No	No
	Semustine	Yes	Yes (N/K)
	Streptozocin	No	No
	Uramustine	No	No
Alkylating agents: Tetrazines	Dacarbazine	Yes	Yes (N/K)
	Temozolomide	No	No
Alkylating agents: Other	Altretamine	Yes	Yes (≤31%)
	Lurbinectedin	Yes	Yes (11%)
	Pipobroman	Muscle cramps (N/K)	No
	Procarbazine	Yes	Yes (N/K)
	Trabectedin	No	No
Antimetabolites: Folic acid analogues	Methotrexate	Neurotoxicity (N/K)	No
	Pemetrexed	No	No

	Pralatrexate	No	No
	Raltitrexed	Asthenia (2-5%)	No
monohydroxyl-substituted urea	Hydroxycarbamide	No	No
	Hydroxyurea	No	No
Antimetabolites: Purine analogues	Cladribine	Neurotoxicity (N/K)	No
	Clofarabine	No	No
	Fludarabine	Asthenia (9% to 65%) and neurotoxicity (N/K)	No
	Mercaptopurine	No	No
	Nelarabine	Yes	Yes (12-21%)
	Pentostatin	Asthenia (10-12%) and neurotoxicity (1-11%)	No
	Rabacfosadine	No	No
	Thioguanine	No	No
Antimetabolites: Pyrimidine analogues	Azacitidine	Asthenia (N/K)	No
	Capecitabine	Paraesthesia (12-21%)	No
	Carmofur	No	No
	Cytarabine	No	No
	Decitabine	Asthenia (15%)	No
	Floxuridine	No	No
	Fluorouracil	Neurotoxicity (N/K)	No
	Gemcitabine	Yes	Yes (15-64%)
	Tegafur+uracil	No	No
	Trifluridine+tipiracil	Asthenia (52%)	No
Corticosteroids (when used as SACT agents)	Dexamethasone	No	No
	Hydrocortisone	No	No
	Methylprednisolone	No	No
	Prednisolone	No	No
	Prednisone	No	No
Cytotoxic antibiotics: Anthracyclines	Aclarubicin	Yes	Yes (N/K)
	Amrubicin	Yes	Yes (1%)
	Daunorubicin	No	No
	Epirubicin	No	No
	Idarubicin	No	No
	Pirarubicin	No	No
	Valrubicin	Asthenia (4%)	No
	Zorubicin	No	No
Cytotoxic antibiotics: Non-Anthracyclines	Bleomycin	No	No
	Dactinomycin	No	No
	Plicamycin	No	No
	Utidelone	Yes	Yes (23%)
Mitotic inhibitors (anti-microtubule	Demecolcine	No	No

agents): Colchicine derivatives			
Mitotic inhibitors (anti-microtubule agents): Taxane derivatives	Cabazitaxel	Yes	Yes (7-18%)
	Docetaxel	Yes	Yes (4-30%)
	Nab-paclitaxel	Yes	Yes (71%)
	Paclitaxel	Yes	Yes (60%)
	Paclitaxel poliglumex	Yes	Yes (19%)
Mitotic inhibitors (anti-microtubule agents): Vinca alkaloids	Vinblastine	Neurotoxicity (N/K)	No
	Vincristine	Yes	Yes (33%)
	Vindesine	Paraesthesia (N/K) and ototoxicity (N/K)	Yes (N/K)
	Vinflunine	Asthenia (N/K)	No
	Vinorelbine	Yes	Yes (20%)
	Vintafolide	Yes	Yes (20%)
Mitotic inhibitors (anti-microtubule agents): Others	Eribulin	Yes	Yes (29-35%)
	Estramustine	No	No
	Ixabepilone	Yes	Yes (62-65%)
Proteasome inhibitors	Bortezomib	Yes	Yes (30-54%)
	Carfilzomib	Yes	Yes (<20%)
	Ixazomib	Yes	Yes (32%)
Topoisomerase (TOP) I inhibitors (camptothecins)	Belotecan	Yes	Yes (6%)
	Etirinotecan pegol	Yes	Yes (3-7%)
	Irinotecan	Asthenia (58-76%)	No
	Topotecan	Asthenia (3-25%)	No
Topoisomerase (TOP) II inhibitors (epipodophyllotoxins)	Amsacrine	Paraesthesia (N/K)	No
	Doxorubicin	No	No
	Etoposide	No	No
	Mitoxantrone	No	No
	Novobiocin	No	No
	Teniposide	Neurotoxicity (<1%)	No

Where there is evidence of associated peripheral neuropathy, evidence of peripheral neurotoxicity is recorded as, 'yes'; where there is no evidence of associated peripheral neuropathy, details of specific neurotoxicity are recorded, where relevant. N/K: not known.

Table S3: Targeted therapy approved for specific types of cancer (N=284)

**Action**	**Agent**	**Evidence of peripheral neurotoxicity**	
		**Any peripheral neurotoxicity**	**Peripheral neuropathy specifically**
Monoclonal antibodies (mAbs): Naked	Alemtuzumab	Yes	Yes (N/K)
	Bermekimab	No	No
	Bevacizumab	No	No

	Cetuximab	Yes	Yes (45%)
	Daratumumab	Yes	Yes (47%)
	Dinutuximab	Yes	Yes (9%)
	Edrecolomab	No	No
	Elotuzumab	Yes	Yes (27%)
	Isatuximab	Yes	Yes (54%)
	Margetuximab	Yes	Yes (16%)
	Mogamulizumab	Guillain–Barré syndrome (N/K)	No
	Naxitamab	Yes	Yes (25-32%)
	Necitumumab	No	No
	Obinutuzumab	No	No
	Ofatumumab	No	No
	Olaratumab	No	No
	Panitumumab	No	No
	Pertuzumab	Yes	Yes (6-42%)
	Prolgolimab	No	No
	Racotumomab	No	No
	Ramucirumab	No	No
	Retifanlimab	Yes	Yes (N/K)
	Rituximab	Asthenia (26%)	No
	Sabatolimab	No	No
	Siltuximab	No	No
	Tafasitamab	No	No
	Toripalimab	Yes	Yes (30%)
	Trastuzumab	Asthenia (5-62%) and paraesthesia (9-48%)	No
	Zolbetuximab	Yes	Yes (≥15%)
Monoclonal antibodies (mAbs): Antibody-drug conjugates	Belantamab mafodotin (conjugated to MMAF)	No	No
	Brentuximab vedotin (conjugated to MMAE)	Yes	Yes (45-81%)
	Datopotamab deruxtecan (conjugated to TOP I inhibitor (DDx))	No	No
	Enfortumab vedotin (conjugated to MMAE)	Yes	Yes (50-67%)
	Gemtuzumab ozogamicin (conjugated to calicheamicin derivative)	No	No
	Ibritumomab tiuxetan (conjugated to a radioactive isotope)	No	No
	Inotuzumab ozogamicin (conjugated to calicheamicin derivative)	No	No
	Loncastuximab tesirine (conjugated to SG3199)	No	No
	Mirvetuximab soravtansine (conjugated to DM4)	Yes	Yes (33-37%)

	Moxetumomab pasudotox (conjugated to Exotoxin A)	No	No
	Ozuriftamab vedotin (conjugated to MMAE)	No	No
	Patritumab deruxtecan (conjugated to TOP I inhibitor)	No	No
	Polatuzumab vedotin (conjugated to MMAE)	Yes	Yes (40-53%)
	Sacituzumab govitecan (conjugated to SN-38)	No	No
	Tisotumab vedotin (conjugated to MMAE)	Yes	Yes (11-38%)
	Trastuzumab deruxtecan (trastuzumab antibody conjugated to deruxtecan)	Yes	Yes (13%)
	Trastuzumab duocarmazine (conjugated to MMAE)	Yes	Yes (26%)
	Trastuzumab emtansine (conjugated to emtansine (DM1))	No	No
Monoclonal antibodies (mAbs): Bispecific	Amivantamab	No	No
	Blinatumomab	Neurotoxicity (65%)	No
	Catumaxomab	No	No
	Elranatamab	Neurotoxicity (59%)	No
	Epcoritamab	No	No
	Glofitamab	Neurotoxicity (N/K)	No
	Margetuximab	Yes	Yes (16%)
	Mosunetuzumab	Neurotoxicity (39%)	No
	Odronextamab	No	No
	Talquetamab	Neurotoxicity (55%)	No
	Tarlatamab	Neurotoxicity (47%)	No
	Teclistamab	Neurotoxicity (15%)	No
	Zanidatamab	No	No
Small molecule: Protein kinase inhibitors	Abemaciclib	No	No
	Abrocitinib	No	No
	Acalabrutinib	No	No
	Afatinib	No	No
	Agerafenib	No	No
	Alectinib	No	No
	Alflutinib	No	No
	Alisternib	No	No
	Almonertinib	No	No
	Alpelisib	No	No
	Alvocidib	No	No
	Apatinib	No	No
	Asciminib	No	No
	AT9283	No	No

	Aumolertinib	No	No
	AV-412	No	No
	Avapritinib	No	No
	Axitinib	Asthenia (21%)	No
	AZD-0424	No	No
	AZD-8330	No	No
	Bafetinib	No	No
	Barasertib	No	No
	Baricitinib	No	No
	Binimetinib	No	No
	Bosutinib	No	No
	Branebrutinib	No	No
	Brigatinib	No	No
	Cabozantinib	No	No
	Capmatinib	No	No
	Capivasertib	No	No
	Catequentinib	No	No
	Cediranib	No	No
	Ceritinib	No	No
	Chiauranib	No	No
	Cobimetinib	No	No
	Copanlisib	No	No
	Crizotinib	No	No
	CYC116	No	No
	Dabrafenib	No	No
	Dacomitinib	No	No
	Danusertib	No	No
	Dasatinib	No	No
	Decernotinib	No	No
	Duvelisib	No	No
	Encorafenib	Yes	Yes (12-62%)
	Ensartinib	No	No
	Entrectinib	Dysesthesia (34%)	No
	Erdafitinib	No	No
	Erlotinib	No	No
	Everolimus	Asthenia (13-33%)	No
	Fedratinib	Asthenia (N/K)	No
	FF-10101-01	No	No
	Flumatinib	No	No
	Fruquintinib	No	No
	Futibatinib	No	No
	Gedatolisib	No	No
	Gefitinib	Asthenia (17%)	No
	Genistein	No	No
	Gilteritinib	No	No
	GSK-2636771	No	No
	Ibrutinib	No	No
	Icotinib	No	No
	Idelalisib	No	No
	Ilorasertib	No	No

Imatinib	Asthenia (12% to 21%)	No
Indirubin	No	No
Infigratinib	No	No
Lapatinib	No	No
Larotrectinib	No	No
Lazertinib	Paraesthesia (35%)	No
Lenvatinib	No	No
Lorlatinib	Yes	Yes (34-47%)
Masitinib	No	No
Merestinib	No	No
Midostaurin	No	No
Mirdametinib	Yes	Yes (21%)
MK-5108	No	No
MKC-1	No	No
MLN8054	No	No
Mobocertinib	No	No
Momelotinib	No	No
Motesanib	No	No
Nab-Sirolimus	No	No
Naporafenib	No	No
Naquotinib	No	No
Neratinib	No	No
Nilotinib	Asthenia (14% to 16%)	No
Nintedanib	No	No
Onvansertib		
Orantinib		
Osimertinib	No	No
Olmutinib	No	No
Pacritinib	No	No
Palbociclib	Yes	Yes (13%)
Parsaclisib	No	No
Pazopanib	No	No
Pemigatinib	No	No
Pelitinib	No	No
Pexidartinib	No	No
PF-03814735	No	No
PF-477736	No	No
Pirtobrutinib	No	No
Ponatinib	Yes	Yes (6-33%)
Pralsetinib	No	No
Prelabrutinib	No	No
Prexasertib	No	No
Quizartinib	No	No
Rabusertib	No	No
Ravoxertinib	No	No
Rebastinib	No	No
Regorafenib	Asthenia (N/K)	No
Repotrectinib	Yes	Yes (49%)
Ribociclib	No	No
Ridaforolimus	No	No

	Rigosertib	No	No
	Ripretinib	No	No
	Rivoceranib	No	No
	Rociletinib	No	No
	Ruxolitinib	No	No
	Selpercatinib	No	No
	Selumetinib	No	No
	Semaxanib	No	No
	Sirolimus	No	No
	Sitravatinib	No	No
	SNS-314	No	No
	Sorafenib	No	No
	Spebrutinib	No	No
	Sunitinib	Asthenia (22% to 34%)	No
	Surufatinib	No	No
	TAK-901	No	No
	Temsirolimus	Asthenia (51%)	No
	Tepotinib	No	No
	Tesevatinib	No	No
	Tirabrutinib	No	No
	Tivozanib	No	No
	Trametinib	No	No
	Tucatinib	No	No
	Umbralisib	No	No
	Vandetanib	No	No
	Vistusertib	No	No
	Vemurafenib	No	No
	Vorolanib	No	No
	Voruciclib		
Small molecule: Other	Abiraterone	No	No
	Adagrasib	Yes	Yes (20%)
	Alitretinoin	No	No
	Anagrelide	Asthenia (23%) and paraesthesia (6%)	No
	Arsenic trioxide	Paraesthesia (33%)	No
	Belinostat	No	No
	Belzutifan	No	No
	Bexarotene	Asthenia (20-45%)	No
	Darinaparsin	No	No
	Decitabine+cedazuridine	Yes	Yes (4-13%)
	Edotreotide	No	No
	Eflornithine	No	No
	Enasidenib	No	No
	Entinostat	No	No
	Epacadostat	No	No
	Glasdegib	No	No
	Idroxioleic acid	No	No
	Imetelstat	No	No
	Inavolisib	No	No

	Ivosidenib	Guillain-Barre syndrome (<1%)	No
	Lonidamine	No	No
	Masoprocol	No	No
	Mifamurtide	Asthenia (13%)	No
	Mitoguazone	No	No
	Navitoclax	No	No
	Niraparib	No	No
	Nirogacestat	No	No
	Oblimersen	No	No
	Olaparib	Asthenia (N/K)	No
	Olutasidenib	No	No
	Omacetaxine mepesuccinate	No	No
	Padeliporfin	No	No
	Pamiparib	No	No
	Panobinostat	No	No
	P-toluenesulfonamide	No	No
	Pelabresib	No	No
	Pixantrone	Asthenia (10% or greater)	No
	Plitidepsin	No	Yes (N/K)
	Porfimer sodium	No	No
	Resminostat	No	No
	Romidepsin	Asthenia (N/K)	No
	Rucaparib	Asthenia (N/K)	No
	Selinexor	Neurotoxicity (25% to 30%)	No
	Sonidegib	No	No
	Sotorasib	No	No
	Suramin	Yes	Yes (≤40%)
	Surufatinib	No	No
	Talazoparib	No	No
	Tazemetostat	No	No
	Tegafur+gimeracil+ oteracil	Yes	Yes (≥10%)
	Temoporfin	No	No
	Tiazofurine	No	No
	Tovorafenib	No	No
	Tucidinostat	No	No
	Veliparib	No	No
	Venetoclax	No	No
	Vismodegib	No	No
	Vorasidenib	No	No
	Vorinostat	No	No
	Vosaroxin	No	No
	Zanubrutinib	No	No
Cell and gene therapy	Lifileucel	No	No
	Nadofaragene Firadenovec	No	No
	Sitimagene ceradenovec	No	No
	Talimogene Laherparepvec	No	No

Fusion proteins	Aflibercept	No	No
	Bifikafusp alfa	No	No
	Calaspargase Pegol	No	No
	Denileukin diftitox	No	No
	Onfekafusp	No	No
	Tagraxofusp	No	No
	Tebentafusp	No	No
Lipophilic compounds	Tanshinone I	No	No
	Tanshinone IIA	No	No
Other protein-based therapies	Asparaginase	Neurotoxicity (N/K)	No
	Crisantaspase	Neurotoxicity (N/K)	No
	Nogapendekin alfa inbakicept- pmln	No	No
	Oportuzumab monatox	No	No
	Pegaspargase	No	No

Where there is evidence of associated peripheral neuropathy, evidence of peripheral neurotoxicity is recorded as, 'yes'; where there is no evidence of associated peripheral neuropathy, details of specific neurotoxicity are recorded, where relevant. N/K: not known.

Table S4: Immunotherapy by class of drug (N=41)

**Action**	**Agent**	**Evidence of peripheral neurotoxicity**	
		**Any peripheral neurotoxicity**	**Peripheral neuropathy specifically**
Autologous cellular immunotherapy	Afamitresgene autoleucel	No	No
Checkpoint inhibitors: PD-1 inhibitors	Cemiplimab	Yes	Yes (11-23%)
	Dostarlimab	Yes	Yes (64%)
	Nivolumab	Yes	Yes (≤12%)
	Pembrolizumab	Yes	Yes (1-67%)
	Pidilizumab *	No	No
	Prolgolimab	No	No
	Prolgolimab + nurulimab	Asthenia (10%)	No
	Serplulimab	No	No
	Tislelizumab	Yes	Yes (N/K)
Checkpoint inhibitors: PD-L1 inhibitors	Atezolizumab	Yes	Yes (33-56%)
	Avelumab	Yes	Yes (N/K)
	Durvalumab	Yes	Yes (61%)
	Sugemalimab	No	No
Checkpoint inhibitors: CTLA-4 inhibitors	Ipilimumab	Yes	Yes (2%)
	Tremelimumab	Yes	Yes (N/K)

Checkpoint inhibitors: LAG-3 inhibitors	Nivolumab+relatlimab	Yes	Yes (<1%)
	Pembrolizumab + favezelimab	No	No
	Cemiplimab + fianlimab	No	No
Chimeric antigen receptor (CAR) T-cell therapy	Axicabtagene ciloleucel (“axi-cel”)	Neurotoxicity (78%)	No
	Brexucabtagene autoleucel (“brexu-cel”)	No	No
	Ciltacabtagene autoleucel (“cilta-cel”)	Neurotoxicity (24-26%)	No
	Idecabtagene vicleucel (“ide-cel”)	Neurotoxicity (28%)	No
	Lisocabtagene maraleucel (“liso-cel”)	No	No
	Obecabtagene autoleucel (“obe-cel”)	Neurotoxicity (64%)	No
	Tisagenlecleucel (“tisa-cel”)	Neurotoxicity (43-71%)	No
Cytokines: Interleukins	Aldesleukin (“Interleukin-2” or “IL-2”)	Asthenia (23%)	No
Cytokines: Interferons	Interferon alfa (IFN-alpha)	Asthenia (N/K) and paraesthesia (13%)	No
	Interferon beta (IFN-beta)	No	No
	Interferon gamma (IFN-gamma)	No	No
	Peginterferon alfa-2a	Yes	Yes (<1%)
	Peginterferon alfa-2b	Yes	Yes (23%) **
Other immune system modulators	Bacillus Calmette-Guérin	Yes	Yes (N/K)
	Histamine dihydrochloride	No	No
	Imiquimod	No	No
	Lenalidomide	Asthenia (≤30%)	No
	Pomalidomide	Yes	Yes (17-18%)
	Sipuleucel-T	No	No
	Talimogene laherparepvec	No	No
	Thalidomide	Yes	Yes (10-54%)
	Tretinoin	Paraesthesia (17-26%)	No

* Pidilizumab: mAb originally thought to bind to the PD-1 immune checkpoint molecule, however, recent evidence suggests that Delta-like 1 (DLL1) is its primary binding target while binding to PD-1 is secondary. ** Specifically, disorder of olfactory nerve. Where there is evidence of associated peripheral neuropathy, evidence of peripheral neurotoxicity is recorded as, ‘yes’; where there is no evidence of associated peripheral neuropathy, details of specific neurotoxicity are recorded, where relevant. N/K: not known.

Table S5: Hormone therapy by class of drug (N=32)

**Action**	**Agent**	**Evidence of peripheral neurotoxicity**

		Any peripheral neurotoxicity	Peripheral neuropathy specifically
Estrogen receptor agonists	Diethylstilbestrol	No	No
	Polyestradiol phosphate	No	No
Estrogenic steroid	Estradiol	No	No
Selective estrogen receptor modulators (SERMs)	Raloxifene	No	No
	Tamoxifen	No	No
	Toremifene	No	No
Selective estrogen receptor degraders (SERDs)	Elacestrant	No	No
	Fulvestrant	Asthenia (6%)	No
Aromatase inhibitors (AIs)	Anastrozole	Asthenia (16-19%)	No
	Exemestane	No	No
	Letrozole	Asthenia (4-34%)	No
Luteinizing hormone-releasing hormone (LHRH) agonists (also called LHRH analogs)	Goserelin	No	No
	Leuprolide	Asthenia (8-18%)	No
	Triptorelin	No	No
Luteinizing hormone-releasing hormone (LHRH) antagonists	Degarelix	No	No
	Relugolix	No	No
CYP17 inhibitors	Abiraterone	No	No
	Ketoconazole	No	No
Androgenic hormones	Fluoxymesterone	Paraesthesia (N/K)	No
Anti-androgens (i.e. androgen receptor antagonists): First generation	Bicalutamide	Asthenia (22%)	No
	Flutamide	No	No
	Nilutamide	No	No
Anti-androgens (i.e. androgen receptor antagonists): Second generation	Apalutamide	No	No
	Darolutamide	No	No
	Enzalutamide	Asthenia (24-51%) and cauda equina syndrome (N/K)	No
Gonadotrophin-releasing hormone (GnRH) agonist	Buserelin	No	No
	Histrelin	No	No
Progestins	Cyproterone acetate	No	No
	Medroxyprogesterone acetate	No	No
	Megestrol acetate	No	No

Adrenolytics	Mitotane	Neurotoxicity (N/K)	No
Somatostatin agonists	Lanreotide	No	No

Where there is evidence of associated peripheral neuropathy, evidence of peripheral neurotoxicity is recorded as, 'yes'; where there is no evidence of associated peripheral neuropathy, details of specific neurotoxicity are recorded, where relevant. N/K: not known.
